# The Impact of* Lactobacillus plantarum* on the Gut Microbiota of Mice with DSS-Induced Colitis

**DOI:** 10.1155/2019/3921315

**Published:** 2019-02-20

**Authors:** Fei Zhang, Yue Li, Xiliang Wang, Shengping Wang, Dingren Bi

**Affiliations:** ^1^State Key Laboratory of Agricultural Microbiology, College of Veterinary Medicine, Huazhong Agricultural University, Wuhan, China; ^2^Key Laboratory of Preventive Veterinary Medicine in Hubei Province, College of Veterinary Medicine, Huazhong Agricultural University, Wuhan, China; ^3^Hunan Institute of Microbiology, Changsha, Hunan, China

## Abstract

The pathogenesis of inflammatory bowel disease (IBD) is due in part to a loss of equilibrium among the gut microbiota, epithelial cells, and resident immune cells. The gut microbiota contains a large proportion of probiotic commensal* Lactobacillus *species; some natural microbiota and probiotics confer protection against IBD. In this study, mice with colitis triggered by dextran sodium sulphate (DSS) were given* Lactobacillus plantarum* orally. We assessed the damage caused by DSS and the therapeutic activity of* L. plantarum*. The colitis triggered by DSS was less severe in the mice that received the* L. plantarum *treatment, which also diversified the microbe species in the colon, enhanced the ratio of Firmicutes to Bacteroidetes, and diminished the relative abundance of* Lactobacillus*. The taxonomic units of greatest diversity in the DSS and* L. plantarum *groups were identified using a linear discriminant and effect size (LEfSe) analysis.* Aliihoeflea* was established to be the genus of bacteria that was affected in the* L. plantarum *group most extensively. In conclusion, gut health was promoted by* L. plantarum*, as it diversified the microbes in the colon and restricted the activity of pathogenic bacteria in the intestine. Moreover, according to the LEfSe analysis, the DSS group was impacted more significantly by gut microorganisms than the* L. plantarum* group, suggesting that* L. plantarum* improved the stability of the intestinal tract.

## 1. Introduction

Crohn's disease (CD) and ulcerative colitis (UC) are collectively known as inflammatory bowel disease (IBD), which manifests itself as a chronic inflammatory relapse of the gastrointestinal tract caused by a range of genetic and environmental factors [1]. A previous report suggested subjects with a genetic predisposition to IBD exhibit abnormal and ongoing inflammatory reactions to the commensal gut microbiome [2]. Research in animal models has also suggested intestinal inflammation is critically dependent on bacterial colonization of the gut, which highlights the importance of the gut microbiota in IBD [3]. A wide range of drug-based treatments are available for IBD, but their effectiveness is moderate and can be accompanied by secondary effects such as toxicity and an increased likelihood of infectious complications [4, 5]. Thus, it is necessary to develop alternative treatments for IBD. Exactly how IBD develops remains uncertain, but there is consensus that the imbalance of homeostasis between the gut microbiota and the mucosal immune system is a major contributor to the disease [6, 7].

Many studies suggest different benefits of probiotics, and IBD could potentially be treated with probiotic supplements [8, 9]. Nevertheless, knowledge about the exact manner in which probiotics protect against IBD remains incomplete. The gut microflora contain an abundance of commensal* Lactobacillus *species, which can rehabilitate homeostasis in intestinal disorders and hence could protect against IBD [10]. As live microorganisms, probiotics have the ability to regulate the composition of the gut microbiota and correct abnormal responses of the mucosal immune system to chronic gut inflammation. Probiotics can also strengthen the gut barrier function by influencing the production of cytokines, stimulating the release of regulatory T cells, and aiding the survival of intestinal cells [11]. Probiotics are advantageous not only from a health perspective, but also from a cost and safety perspective. The only drawback of this strategy is that a comprehensive understanding of how probiotics exert their health effects has not yet been achieved [12].

We conducted an experiment in mice with colitis induced by dextran sodium sulphate (DSS) to determine the impact of* Lactobacillus plantarum *on gut inflammation and whether the effects of this bacterium were correlated with the immune response and gut microbiota.

## 2. Materials and Methods

### 2.1. Animals

The animal experiments were approved by the Medical Ethics Committee of Huazhong Agricultural University, and we complied with their guidelines while conducting the experiments. The Hubei Province Centre for Disease Control and Prevention (Wuhan, China) provided 20 specific pathogen free (SPF) ICR mice of female sex. The mice were 8-10 weeks of age and weighed 20±2.1 g. The conditions under which the mice were kept included an SPF environment with alternating 12 h of light and 12 h of darkness. There were no restrictions on water or food. The mice were allowed to become accustomed to the laboratory conditions for 7 d prior to the experiments.

### 2.2. Colitis Induced by DSS and the Structure of the Experiments

The mice were separated into two groups according to a completely randomized design. The groups were a DSS group and an LPZ group, which were, respectively, given a basal diet [13] and a basal diet enriched with 2 x 10^10^ CFU/kg* L. plantarum *for one week. Colitis was triggered by giving all the mice 5% DSS (MW 36-50 kDa, Kayon Biological Technology Co. Ltd.) in the drinking water on day 8 [13, 14]. The substitution of DSS was conducted daily for one week. Upon completion of the experiments, the average weight gain per day was determined by weighing the mice on the morning of day 15; the mice were anesthetized intraperitoneally and then killed. The measurement of colon length and weight was conducted in line with an earlier study [13]. During the experiment process, the modifications in body weight and disease activity index (DAI) were measured every day to determine how severe the colitis was according to a previously published method [13, 14]. Briefly, the mice were also subjected to evaluation in terms of body weight loss (score: 0 = none; 1 = 0-5%; 2 = 6-10%; 3 = 11-15%; 4 = 16-20%; 5 = 21-25%; and 6 = 26-30%), stool consistency (score: 0 = normal stool; 1 = soft stool; and 2 = liquid stool), and rectal bleeding (score: 0 = negative fecal occult blood; 1 = positive fecal occult blood; and 2 = visible rectal bleeding).

### 2.3. Histopathological Analysis

The procedures of extraction and fixation in 10% formalin of the terminal colon were conducted. Hematoxylin and eosin were used for preparation and staining of the sections embedded in paraffin for a subsequent histology-based assessment and scoring of epithelial loss (0 = no loss; 1 = 0-5% loss; 2 = 5-10% loss; and 4 = more than 10% loss), crypt damage (0 = no damage; 1 = 0-10% damage; 2 = 10-20% damage; and 3 = more than 20% damage), reduction in the number of goblet cells (0 = none; 1 = mild; 2 = moderate; and 3 = severe), and inflammatory cell infiltration (0 = none; 1 = mild; 2 = moderate; and 3 = severe). These scores were summed to obtain the overall score.

### 2.4. Enzyme-Linked Immunosorbent Assay

Sandwich enzyme-linked immunosorbent assays (ELISA Ready-SET-GO, eBioscience, CA, USA) were conducted for the purpose of measuring TNF-*α*, IL-1*β*, IL-6, IL-10, and IL-17A levels in the colon tissues. Color development was achieved with horseradish peroxidase-avidin. An ELISA microplate reader (Molecular Devices, Sunnyvale, CA, USA) permitted the measurement of the absorbance at 405 nm.

### 2.5. DNA Purification and Amplification

Prior to conducting the process of extracting DNA, the samples were kept in storage at a temperature of -80°C. The QIAamp DNA Stool Mini Kit (QIAGEN, Hilden, Germany) was used in keeping with the manufacturer's guidelines to extract the DNA from the 200 mg samples. The samples were run on a 1.0% agarose gel to verify how concentrated and pure the DNA was. The general bacterial primers 515F 5′-GTGCCAGCMGCCGCGGTAA-3′ and 926R 5′-CCGTCAATTCMTTTGAGTTT-3′ were used to amplify 16S rRNA genes by carrying out a polymerase chain reaction (PCR). According to the manufacturer's guidelines regarding overhang sequences, the two primers included the primer of the Illumina 5′ overhang sequence and dual barcodes for the two-step construction of the amplicon library. The reaction volumes (25 *μ*L) comprising 1-2 *μ*L DNA template, 250 mM dNTPs, 0.25 mM of each primer, 1X reaction buffer, and 0.5 U Phusion DNA Polymerase (New England Biolabs, USA) were used to undertake the first PCR reactions. The PCR conditions involved an initial 2 min denaturation at 94°C, followed by 30 cycles of 30 s denaturation at 94°C, 30 s annealing at 56°C, and a 30 s extension at 72°C. These conditions were followed by a final 5 min extension at 72°C. The adaptors and 8 base barcodes were added to either end of the 16S amplicons via eight-cycle PCR reactions of the second-step PCR. The cycling conditions involved an initial 2 min denaturation at 94°C, followed by 30 cycles of 30 sec denaturation at 94°C, 30 sec annealing at 56°C, and a 30 sec extension at 72°C. These conditions were followed by a final 5 min extension at 72°C. A DNA gel extraction kit (Axygen, China) and FTC -3000 TM real-time PCR were, respectively, used for purification and quantification of the barcoded PCR products before library pooling.

### 2.6. DNA Sequencing and Bioinformatic Analysis

A HiSeq Rapid SBS Kit v2 (Illumina; Tiny Gene Bio-Tech (Shanghai) Co., Ltd) was used for the sequencing of the libraries based on 2*∗*250 bp paired-end sequencing on the HiSeq platform. The barcode enabled the demultiplexing of the raw fastq files, and base pairs of poor quality were eliminated by running PE reads for every sample via Trimmomatic (version 0.35) and using the parameters SLIDINGWINDOW: 50:20 and MINLEN: 50. The Flash program (version 1.2.11) was subsequently used with default parameters to further integrate the trimmed reads. The screen.seqs command was applied alongside the filtering parameters maxambig=0, minlength=200, maxlength=580, and maxhomop=8 to eliminate contigs of poor quality. The software packages mothur (version 1.33.3), UPARSE (usearch version v8.1.1756, http://drive5.com/uparse/), and R (version 3.2.3) were all used for the purposes of analysis of the 16S sequences. Meanwhile, the UPARSE pipeline (http://drive5.com/usearch/manual/uparse_pipeline.html) permitted the grouping of demultiplexed reads at 97% sequence identity into operational taxonomic units (OTUs). The classify.seqs command in mothur enabled the allocation for taxonomy of the OTU representative sequences against the Silva 119 database with a confidence score equal to or greater than 0.8. NCBI helped to determine OTUs, from phylum to species. mothur permitted the determination of the Shannon, Simpson, Chao, and ACE indices and rarefaction curves for the alpha-diversity analysis, while R was used for plotting those curves. Moreover, mothur also enabled the determination of the weighted and unweighted UniFrac distance matrix for the beta-diversity metrics and principal coordinate analysis (PCoA) and a tree by R facilitated visualization. R was used for both determination and visualization of the Bray-Curtis metrics.

### 2.7. Statistical Analysis

GraphPad Prism (V.6.0 for Windows; GraphPad Software) based on the Student's* t-*test was used for every statistical analysis. The experimental values are expressed as mean ± standard error of the mean (SEM). Statistical significance is given by a* P *value of less than 0.05.

## 3. Results

**Figure 1:**
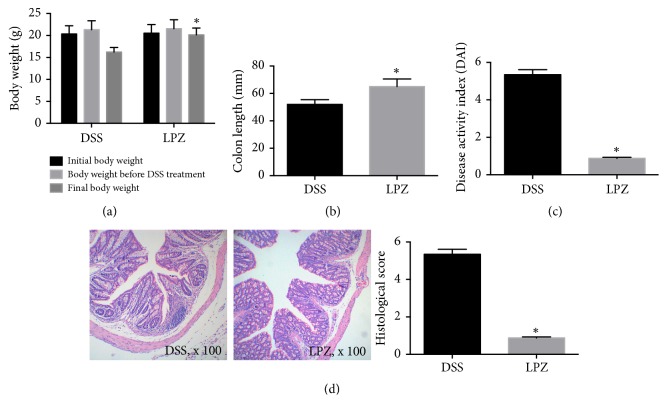
Effects of* L. plantarum* on (a) the body weight, (b) colon length, (c) disease activity index, and (d) histological score. n=10. *∗* indicated* P* < 0.05 for comparison of the LPZ and DSS groups.

To determine the impact of* L. plantarum *on the manner in which colitis developed and how severe it became, the weight and colon length of mice from both experimental groups were measured (Figures 1(a) and 1(b)). The LPZ group had a significantly higher body weight and a longer colon length than the DSS group (*P *< 0.05). Meanwhile, the LPZ group had a significantly lower DAI (Figure 1(c)) and histological score (Figure 1(d)) than the DSS group (*P* < 0.05).

**Figure 2:**
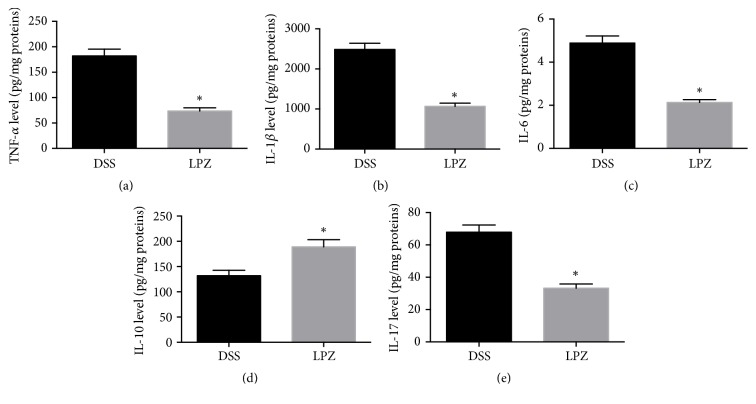
Effects of* L. plantarum* on the (a) TNF-*α*, (b) IL-1*β*, (c) IL-6, (d) IL-10, and (e) IL-17 of the colonic tissues. N=8, *∗* indicated* P* < 0.05 for comparison of the LPZ and DSS groups.

An ELISA was used to evaluate the protection conferred by* L. plantarum *against colitis through modulation of inflammatory cytokines (Figure 2). In comparison to the DSS group, the levels of IL-1*β*, IL-6, IL-17, and TNF-*α* were decreased in the LPZ group. The level of IL-10 was elevated in the experimental group administered* L. plantarum *(*P* < 0.05).

**Figure 3:**
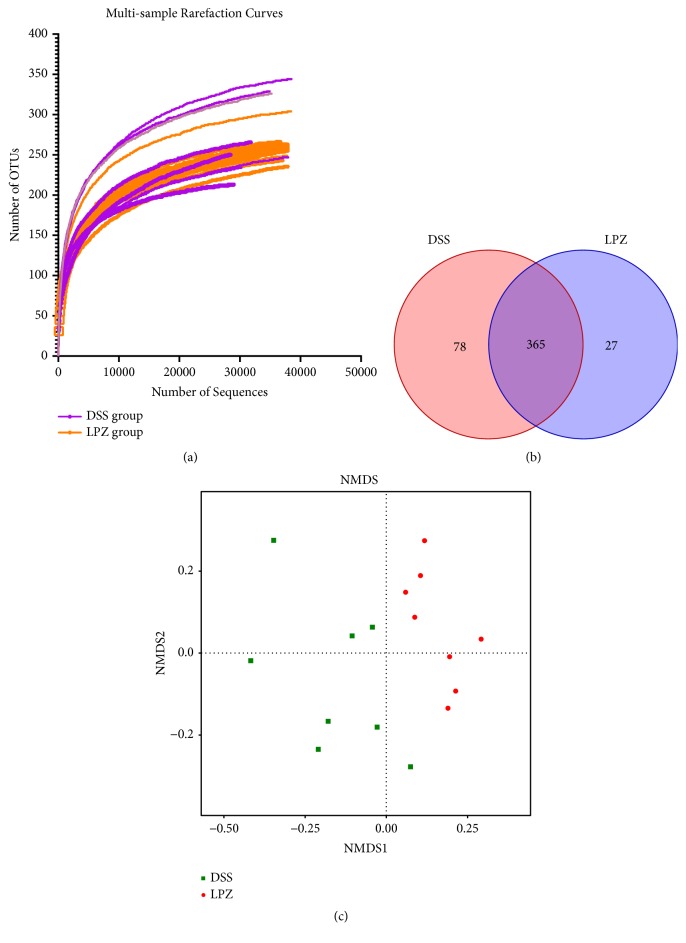
Comparison of the colonic microbial sequences and NMDS analysis between the DSS and LPZ groups. (a) Rarefaction curves show the numbers of unique OTU for each sample. (b) The Venn diagram depicts OTUs that were unique to the 8 mice in the DSS and LPZ groups. (c) The NMDS analysis of the differences between the DSS and LPZ groups. The samples are separated into different parts of the plot, indicating differences between groups or within groups. N=8.

The v3-v4 regions of 16S rRNA obtained from the fecal samples of the colon were sequenced. The rarefaction curves of the numbers of observed OTUs per sample indicated the mean numbers of observed OTUs were approximately 28,000 sequence reads (Figure 3(a)). Out of the 580 bacterial species-level OTUs found in this study, only 365 (63%) were shared (Figure 3(b)). Nonmetric multidimensional scales (NMDS) were used to detect the relationship among colonic eco-communities to determine whether OTUs identified using the Kruskal-Wallis (KW) filters differentiated between the DSS and LPZ mice. The identified taxa successfully divided the mice into two different groups, and only 1 of the 8 mice in the DSS group clustered with the LPZ group (Figure 3(c)).

**Figure 4:**
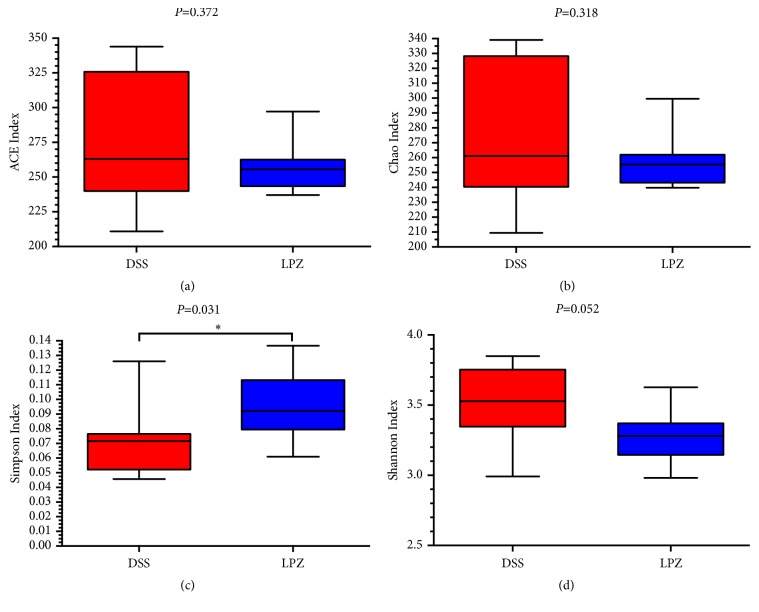
Phylogenetic diversity of colonic microbiota between the DSS and LPZ groups. Box plots indicate microbiome diversity differences of (a) ACE diversity, (b) Chao diversity, (c) Simpson diversity, and (d) Shannon diversity between the DSS and LPZ groups. N=8, *∗* indicates* P* < 0.05.

The colonic microbial diversity was measured by ACE diversity (Figure 4(a)), Chao diversity (Figure 4(b)), Simpson diversity (Figure 4(c)), and Shannon diversity (Figure 4(d)) between the DSS and LPZ groups.* L. plantarum* increased the Simpson index in relation to the mice in the DSS group (*P* < 0.05), but there were no significant effects on the other indices.

**Figure 5:**
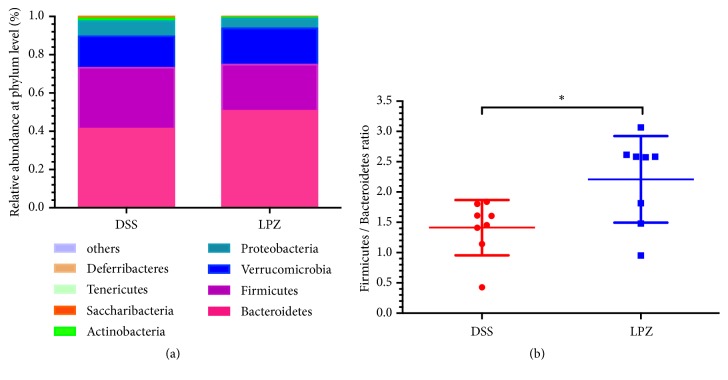
Analysis of microbial composition at the phylum level. (a) Phylum level microbial changes in the colon in the DSS and LPZ groups. (b) Ratio of Bacteroidetes to Firmicutes in the colon of mice from the two groups. N=8, *∗* indicates* P* < 0.05.

The bacteria of the Bacteroidetes, Firmicutes, Verrucomicrobia, and Proteobacteria were predominant at the phylum level, accounting for more than 97% of the total microbial composition (Figure 5(a)). In the DSS and LPZ groups the respective proportions of Bacteroidetes were 41.09% and 50.36%, and for Firmicutes the proportions were 31.78% and 24.09%. The ratio of Firmicutes to Bacteroidetes was increased in LPZ group compared to the DSS group (*P*<0.05) (Figure 5(b)).

**Figure 6:**
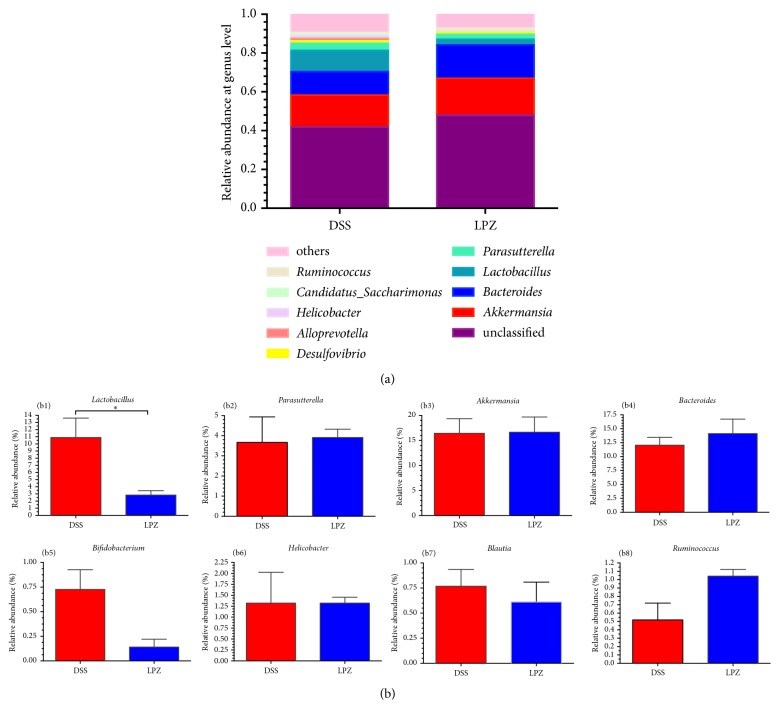
Analysis of the microbial composition at the genus level. (a) Genus-level microbial changes in the colon of the DSS and LPZ groups. (b) Comparison of genus-level microbiota in the DSS and LPZ groups: (b1)* Lactobacillus*; (b2)* Parasutterella*; (b3)* Akkermansia*; (b4)* Bacteroides*; (b5)* Bifidobacterium*; (b6)* Helicobacter*; (b7)* Blautia*; and (b8)* Ruminococcus*. N=8, *∗* indicates* P* < 0.05.

Figure 6 shows the top 10 generic-level microbes of relative abundance in the DSS and LPZ groups. The top five genera in the control group were* Akkermansia* (16.45%),* Bacteroides* (12.05%),* Lactobacillus* (10.89%),* Parasutterella* (3.67%), and* Desulfovibrio* (1.25%); in the LPZ group the top five strains were* Akkermansia* (19.02%),* Bacteroides* (17.23%),* Lactobacillus* (2.84%),* Parasutterella* (2.45%), and* Desulfovibrio* (1.06%). The LPZ treatment had a negative effect on the relative abundance of* Lactobacillus *in comparison to the DSS group, with an 8.05% reduction (*P*<0.05).

**Figure 7:**
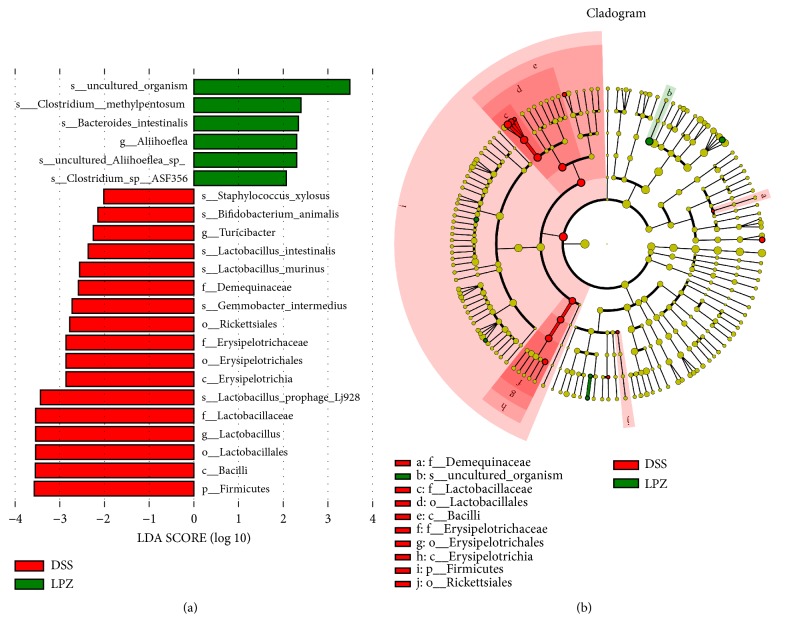
LEfSe and LDA analyses based on OTUs characterized the microbiomes of the DSS and LPZ groups. (a) LDA scores show the significant bacterial differences between the LCT and HT (log LDA >2.0; n = 8). (b) Cladogram using the LEfSe method shows the phylogenetic distribution of the colonic microbes associated with the mice administrated DSS (green) and the mice treated with* L. plantarum* (red).

To identify the specific bacteria in the DSS and LPZ groups, the colonic microbial differential species were analyzed using the linear discriminant analysis (LDA) effect size (LEfSe) based on a nonparametric factorial KW and rank test.  Figure 7 shows the species with significant differences, indicated by an LDA score greater than 2.0, which reflects the degree of influence of a species with a significant difference between the groups. A pairwise comparison between the gut microbiota of the DSS and LPZ groups revealed that, at the genus level, the DSS treatment increased the abundance of* Aliihoeflea* and increased* Clostridium methylpentosum* and* Bacteroides intestinalis*; uncultured* Aliihoeflea sp* and* Clostridium sp ASF356* were increased at the species level. Compared to the DSS treatment, there were more diverse changes in the structure of the colonic microbiota. At the phylum level, the oral administration of* L. plantarum* enriched the amount of Firmicutes and increased Erysipelotrichia and Bacilli at the class level. In addition,* L. plantarum *treatment significantly increased the abundance of* Turicibacter* and* Lactobacillus* at the genus level and* Staphylococcus xylosus*,* Bifidobacterium animalis*,* Lactobacillus intestinalis*,* Lactobacillus murinus*,* Gemmobacter intermedius*, and* Lactobacillus prophage Lj928 *at the species level.

## 4. Discussion

IBD manifests as mucosal and systemic inflammation occurring primarily in the large intestine, as in the case of UC, or at any site within the gastrointestinal tract as in the case of CD. The reason for the occurrence of such an inflammatory response is mucosal immune intolerance and especially the disruption of the equilibrium between the anti-inflammatory cytokine IL-10 and other cytokines.

A wide range of IBD treatments exist, but treatment unresponsiveness and occurrence of side-effects continue to be experienced by some patients [15]. Among the latest promising IBD treatments is therapy involving the oral administration of lactic acid bacteria [16, 17]. However, it is still unclear exactly how particular action mechanisms and the therapeutic roles of bacteria are correlated, so the clinical feasibility of this therapy is under debate [18, 19]. We used a mouse model of colitis triggered by DSS to investigate the use of the most popular probiotic,* L. plantarum*, in IBD treatment. The mice treated with* L. plantarum *did not lose weight or exhibit a reduced colon length. Furthermore, in comparison to the DSS group, the* L. plantarum *group exhibited less pronounced DAI and histological alterations.

IL-10 production was also stimulated by* L. plantarum *directly, but the IL-17 production was inhibited. Recent evidence points to the involvement of IL-17A in the fibrosis of the lungs, liver, and heart [20–22]. Conversely, inflammatory models with suppression of IL-17 revealed the amelioration of fibrosis, while pulmonary fibrosis models showed that cardiac fibroblasts were encouraged by IL-17A to proliferate and migrate [23]. Such findings highlight the fact that fibrosis depends on IL-17 and Th17 cells. In addition to providing protection from the key proinflammatory cytokine TNF-*α* [24], IL-10 also regulates chronic intestinal inflammation, so colitis of high severity occurs when low IL-10 levels are associated with intestinal endoplasmic reticulum (ER) stress [24, 25]. Meanwhile, evidence has been put forth that IL-10 production in colitis can be stimulated by certain probiotics [26, 27]. Some support exists for the idea that colitis attenuation can be achieved by certain* Lactobacillus *strains through an increase of regulatory T cells (Tregs) in colonic tissues [28]. Nevertheless, the mechanism of the impact of probiotics and IL-10 on ER stress remains unknown, and more research needs to be conducted to determine precisely how* L. plantarum *exerts its effects. Our results imply* L. plantarum *has potential for use as an effective immunomodulator in IBD and could make a notable contribution to IBD treatment.

The value of probiotics for preventing and treating gastrointestinal disorders is gaining recognition [8]. As live microorganisms, probiotics can be beneficial to host health, provided they are administrated in suitable concentrations. They modulate the proinflammatory and anti-inflammatory cytokines produced by the immunocytes in the gut and thus contribute to the maintenance of homeostasis in the gut microbiota [29, 30]. There are a number of probiotic strains that could be beneficial for IBD, but the clinical use of probiotics has produced incongruous outcomes [31]. Studies examining how probiotics acted against inflammation found NF-*κ*B activation and the expression of inflammatory cytokines in mice with colitis were both hindered by probiotics [32, 33].

Health and wellbeing are dependent on beneficial symbionts and commensals having a mutualistic relation; otherwise dysbiosis and, eventually, disease occur [34]. There is a high degree of complexity to such a “symbiotic ecosystem,” and the lumen and external mucosal layer of the colon contain the greatest aggregation of microorganisms. We investigated the effects of* Lactobacillus plantarum* on colonic microorganisms in a DSS-induced colitis mouse model. The* L. plantarum* treatment increased the colonic microbial diversity and Firmicutes/Bacteroidetes ratio but reduced the relative abundance of* Lactobacillus*. The LEfSe and LDA analyses were used to determine the most diverse taxonomic units in the DSS and the LPZ groups. The bacterium with the greatest influence on the LPZ group at the genus level was* Aliihoeflea*. The bacteria affecting the DSS group were diverse and distributed in various taxon units, such as Firmicutes at the phylum level and* Turicibacter* and* Lactobacillus* at the genus level. The gut microbiota is made more resilient by microbial diversity, contributing significantly to health and wellbeing [35]. The condition of the human gut microbiota has been demonstrated to be reflected in the ratio of Firmicutes to Bacteroidetes [36]. This ratio was decreased in mice with diabetes [37] and in some CD and UC patients, alongside a relative proliferation of proteobacteria [38]. A greater diversity in microbial species results in a more diverse functional response, defined as the level of sensitivity variation to ecosystem modifications exhibited by a species in a community contributing to the same ecosystem function. For instance, when an environmental disruption impacts an abundant species, a less abundant species fulfilling a similar function can take on the role of the abundant species when functional response is suitably diverse [39, 40]. To maintain gut health, beneficial bacteria must be promoted, and pathogenic bacteria must be minimized in the gut microbiota.

There are also some shortcomings of this study. The findings indicate* L. plantarum *reduced the expression of proinflammatory cytokines, thus having a positive impact on colitis. The mechanisms of the inflammation regulation remain unknown. The study also focused solely on the preventive action of* L. plantarum *and not on its therapeutic action. Future research should address these issues and the mechanisms through which the progress of colitis is slowed.

To summarize,* L. plantarum *significantly contributed to the suppression of the inherent production of proinflammatory cytokines during the development of colitis and is likely to ameliorate the pathophysiology of colitis triggered by DSS.* L. plantarum *improved the intestinal tract stability as the impact of intestinal microorganisms was less extensive in the* L. plantarum *group than the DSS group. In conclusion,* L. plantarum *might be effective for managing colitis symptoms and have potential as an effective IBD therapeutic agent.

## Data Availability

The data used to support the findings of this study are available from the corresponding author upon request.
